# Magnetically driven preparation of 1-D nano-necklaces capable of MRI relaxation enhancement†

**DOI:** 10.1039/d3na00137g

**Published:** 2023-05-24

**Authors:** Aaron M. King, Teresa Insinna, Connor J. R. Wells, Isabel A. Raby, Yurii K. Gun'ko, Gemma-Louise Davies

**Affiliations:** a Department of Chemistry, University College London 20 Gordon Street London WC1H 0AJ UK; b School of Chemistry, Trinity College Dublin Dublin 2 Ireland gemma-louise.davies@ucl.ac.uk

## Abstract

We report a novel magnetically-facilitated approach to produce 1-D ‘nano-necklace’ arrays composed of 0-D magnetic nanoparticles, which are assembled and coated with an oxide layer to produce semi-flexible core@shell type structures. These ‘nano-necklaces’ demonstrate good MRI relaxation properties despite their coating and permanent alignment, with low field enhancement due to structural and magnetocrystalline anisotropy.

## Abstract 2

We report a novel magnetically-facilitated approach to produce 1-D ‘nano-necklace’ arrays composed of 0-D magnetic nanoparticles, which are assembled and coated with an oxide layer to produce semi-flexible core@shell type structures.

## Graphical abstract

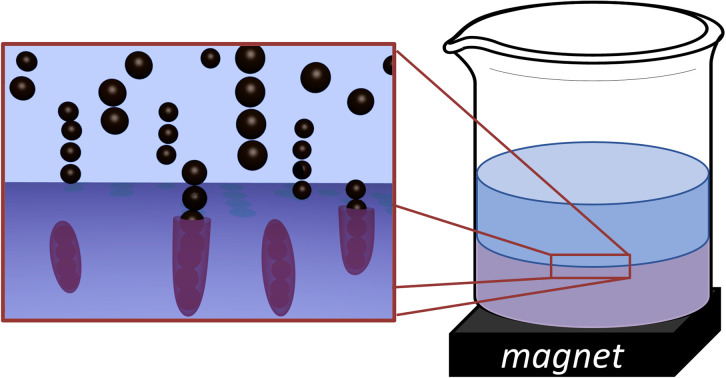

Magnetic 1-dimensional (1-D) nanostructures, which have one dimension outside of the nanoscale (<100 nm) range, typically consist of nanowires, nanotubes, nanofibers and aligned arrays of nanoparticles. They have become increasingly popular over the past two decades, due to their wide-ranging potential in magnetic recording, lithium ion batteries, sensors, catalysis and medicine.^1–4^ There has been some evidence that 1-D structures can outperform their nanoparticle (or 0-dimensional (0-D)) counterparts in certain scenarios. This is due to their high aspect ratio which translates into magnetic, optical, electronic or chemical anisotropy, providing unique beneficial features, such as unusual magnetic properties or enhanced electronic and ionic transport.^5–7^ Such isotropic properties are particularly attractive for biological applications, where the elongated shape of magnetic 1-D materials can, for example, enhance magnetic separation, cell binding and interaction, due to multivalent cellular interactions and higher magnetic moments than 0-D equivalents, providing exceptional cellular recognition and internalisation, and efficient magnetic removal.^7–9^ Increased shape anisotropy and improved dipole–dipole interactions can boost magnetic hyperthermia behaviour.^10^ As contrast agents for magnetic resonance imaging (MRI), 1-D assemblies of nanostructures, nanowires and nanoworms offer superior signal enhancement.^11–15^ This is due to increased magnetisation and local magnetic field strength of the 1-D structures producing boosted proton relaxation rate enhancement compared to their 0-D analogues. This signal enhancement behaviour is predominantly due to the increased magnetic anisotropy of these 1-D structures or assemblies. Some 1-D materials have also reported long blood circulation half-lives, with low macrophagic uptake due to their aspect ratio and alignment in the direction of blood flow.^7^ This is an important consideration for their biomedical application, particularly in *in vivo* imaging and targeting, where long blood circulation is vital.

Efficient and reproducible routes to the fabrication of 1-D magnetic nanostructures have hence become a matter of high importance. The most common approaches include lithography,^16^ template-assisted synthesis,^2,8,17^ electrospinning,^18^ solvo/hydrothermal processing,^19^ and vapour-solid growth.^20^ Self-assembly has become popular recently *via* the use of molecular linkers or exploiting the properties of nanoparticles, such as charge or stimulus-response, as building blocks towards 1-D nanostructures.^21,22^ Utilising magnetic nanoparticles as building blocks is an intriguing approach to producing 1-D nanostructures. One such approach relies on weakly ferromagnetic particles possessing a permanent magnetic dipole to spontaneously align in the absence of an applied magnetic field.^23^ Alternatively, nanowires assembled from nanoparticle building blocks can be produced *in situ* through the application of an external magnetic field during material synthesis, as a result of magnetic dipoles forming aligned chains of nanoparticles which are permanently fused by heat during synthesis.^24,25^ Magnetic fields can also direct assembly of magnetic nanoparticles after their initial preparation, followed by coating (usually with a polymer) to retain their chain-like structure.^26–29^

An extrapolation of these approaches could lie in generating chemical reactions at particle surfaces during their physical alignment to result in permanent 1-D structures; for example, stimulating a chemical reaction at material surfaces at the point when 0-D particles are aligned into 1-D structures. This has the potential to provide control over the alignment, the resulting aspect ratios of 1-D structures, as well as the final surface chemistries. Herein, we present a straight-forward synthetic strategy based on magnetic triggering of trans-phase reactions in bi-layer aqueous-organic solvent systems to produce permanent 1-D assemblies of oxide coated magnetic nanoparticles (Scheme 1). Our approach involves the use of a magnetic field (a permanent magnet) to align magnetic nanoparticles into flexible 1-D arrays in an aqueous phase, followed by the straight-forward phase transfer of the arrays into an organic phase, containing reactive metallorganic precursors. This process triggers the hydrolysis of metallorganic precursors at the nanoparticle surface and *in situ* coating of magnetic arrays with an oxide layer, resulting in the production of aligned oxide-coated 1-D nanostructures, which retain their magnetic capabilities. We further demonstrate that the particles retain strong relaxation properties of use in magnetic resonance imaging (MRI).

**Scheme 1:**
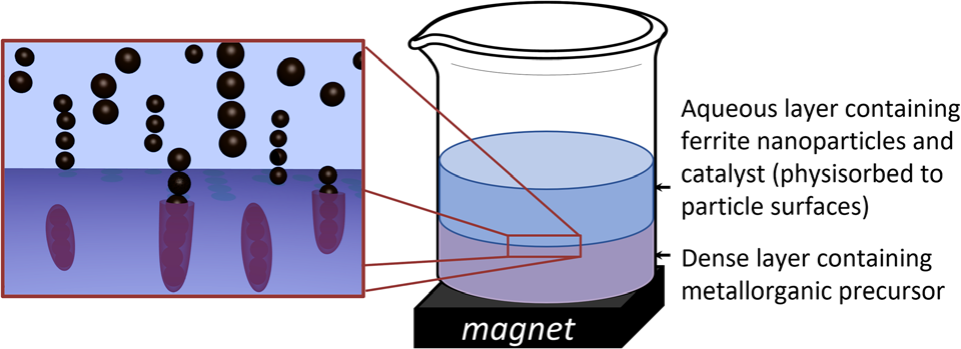
Schematic representation of magnetic phase-transfer approach in the production of oxide-coated magnetic 1-D ‘nano-necklace’ constructs. Top (aqueous) layer contains stabilised magnetic nanoparticles and catalyst species (herein, NH_4_OH); bottom (dense) layer (dichloromethane) contains monomer species (tetraethoxysilane). Alignment of stabilised magnetic nanoparticles into 1-D arrays occurs due to the presence of the magnetic field; as particles move towards the magnet, they move into the lower phase containing a monomer species. Physisorbed catalyst species on the magnetic particle surfaces triggers a hydrolysis and condensation reaction, resulting in the formation of an oxide shell around the nanoparticles (herein shown as a purple ‘coating’), leading to the formation of oxide-coated arrays of magnetic ‘nano-necklaces’.

Cobalt ferrite (CoFe_2_O_4_) nanoparticles stabilised with a negatively charged polyelectrolyte (poly(sodium-4-styrene)sulfonate, PSSS) were initially prepared according to published procedures (see Experimental, ESI†).^30^ Transmission electron microscopy (TEM) confirmed the formation of quasi-spherical nanoparticles comprised of smaller CoFe_2_O_4_ cores (*d*_core_ = 55.3 ± 13.6 nm) (Fig. 1 and S1, ESI†). The Raman modes shown in Fig. S2, ESI,† 680 cm^−1^, 617 cm^−1^, 475 cm^−1^, and 300 cm^−1^ are characteristic of the cubic inverse spinel structure of the CoFe_2_O_4_.^31^ Powder X-ray diffraction (P-XRD), further confirmed this, with reflection peaks observed at 13.7°, 16.1°, 19.4°, 23.8°, 25.3°, and 27.6° 2*θ*. These can easily be indexed to (220), (311), (400), (422), (511) and (440) planes respectively, correlating well with that reported for cubic cobalt ferrite (Fig. S3, ESI†). Vibrating sample magnetometry (VSM) measured the magnetisation saturation of the stabilised nanoparticles to be 66.2 emu g^−1^ at 20 000 Oe (2.0 T). The particles exhibited narrow hysteresis, with a coercivity of 53.3 Oe, indicating slight ferromagnetic behaviour (Fig. S4, ESI†). This is concurrent with previous observations of similar materials.^30,32^

**Fig. 1:**
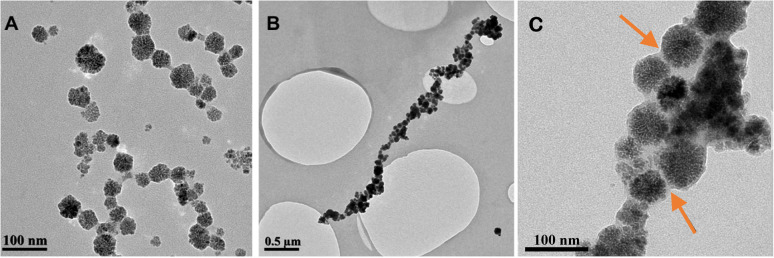
Transmission electron microscope (TEM) images of (A) PSSS-stabilised CoFe_2_O_4_ parent particles, and silica-coated CoFe_2_O_4_ nano-necklaces produced using magnetic trans-phase attraction method at low, (B), and high magnification, (C). Silica coating highlighted by arrows.

We and others have previously reported the *in situ* stabilisation of magnetic particles using polyelectrolytes,^30,33,34^ wherein the polyelectrolytes act not only as a colloid stabiliser, but also as a templating species, providing sites along the negatively charged backbone for the particles to seed during preparation. As a consequence of this templating behaviour, particles can form organised 1-D linear arrays upon application of a permanent external magnetic field, as observed in TEM (Fig. S1b, ESI†), behaviour not observed when magnetic particles are prepared in the absence of PSSS (Fig. S1a, ESI†).^30,33,35,36^ The formation of these (reversible) linear arrays makes them useful precursors for the formation of the ‘permanent’ nano-necklace constructs herein.

Using a unique experimental setup, the stable aqueous colloidal nanoparticles (with added ammonium hydroxide catalyst) were magnetically attracted through a second, higher density, solvent phase (dichloromethane, DCM) containing moisture sensitive precursors (tetraethoxysilane, TEOS), as depicted in Scheme 1. In the presence of a magnetic field, the particles align into 1-D arrays. As the particles, together with an electrostatically-adsorbed surface layer of catalyst (NH_4_OH) are attracted towards the magnet, passing into the second phase, a hydrolysis and condensation reaction of tetraethoxysilane is initiated by the catalyst at the surface of the aligned magnetic nanoparticles, resulting in their coating with a thin layer of silica (inset, Scheme 1). The coated nanoparticle arrays collect at the bottom of the flask close to the magnet, facilitating their easy removal and purification prior to characterisation.

Prior to coating, the PSSS-stabilised CoFe_2_O_4_ nanoparticles exist as spherical particles, which are well-dispersed in suspension and exhibit no 1-dimensional alignment in the absence of a magnetic field (Fig. S1b, ESI†). After trans-phase magnetic treatment, the nanoparticles exhibit a quasi-linear somewhat branched morphology, with a silica shell coating the external surface of the particles (Fig. 1b and c). The prepared 1-D assemblies had lengths of 2.15 ± 1.4 μm. A shell of silica can clearly be observed in Fig. 1c. FTIR additionally confirmed the presence of the silica shell, with a band between 1100 and 990 cm^−1^, typical of asymmetric Si–O–Si stretches (Fig. S5, ESI†).^37^ Raman and XRD confirmed the same modes and reflections as expected for the cobalt ferrite phase, with minimal changes (Fig. S2 and S3, ESI†). A magnetisation saturation of 53.6 emu g^−1^ for the nano-necklace sample was slightly lower than the parent particles due to the presence of non-magnetic silica shell contributions (Fig. S4, ESI†).

Variation of the concentration of base catalyst (see Experimental, ESI†), resulted in a thicker layer of silica (20.1 ± 3.8 nm) when NH_4_OH concentration was increased to 2.5 M (silica shell thickness of sample in Fig. 1 was 4.7 ± 1.5 nm). The increased concentration of ammonium hydroxide additionally resulted in increased free silica present throughout (Fig. S6, ESI†), whereas with lower NH_4_OH concentrations (0.09 M), thinner and less uniform coatings were produced, with insufficient silica shell to maintain the 1-D assemblies, resulting in non-uniform aggregates (Fig. S6, ESI†). The use of a stronger magnet during preparation (45.0 kg pull strength compared to 16.3 kg pull strength in other experiments, see Experimental, ESI†) resulted in the formation of more branched chains of silica coated necklaces with more parallel rows of particles, and lengths and widths of 4.18 ± 1.84 μm and 0.14 ± 0.16 μm, respectively (Fig. S7, ESI†). These larger structures were a result of the stronger magnetisation of the parent particles in response to the increased magnet strength, and hence stronger magnetic attractions between particles leading to ‘bundles’ of chains, similar behaviour has been observed previously in literature.^29,38^ Unfortunately, due to their large sizes, these samples were not colloidally stable enough for further measurement.

Control experiments were carried out using our experimental setup in the absence of a magnetic field. No movement of nanoparticles through the solvent phase interface occurred, even over extended periods of time (up to 24 h), therefore no coating or 1-D nanostructures were formed. This confirms that coated 1-D arrays of magnetic nanoparticles are only formed due to the presence of a magnetic field triggering both alignment and movement of the particles through the solvent bi-layer to produce an oxide coating at the surface of magnetic nanostructures. The use of non-stabilised CoFe_2_O_4_ nanoparticles additionally did not lead to the formation of nano-necklaces, due to the significant (non-directed) aggregation of the particles under the influence of a magnetic field (Fig. S1, ESI†).

Single field ^1^H relaxation measurements (23 MHz) of parent stabilised CoFe_2_O_4_ and nano-necklaces were in line with relaxivities observed for similarly prepared stabilised cobalt ferrite nanoparticles, with high *r*_2_/*r*_1_ values indicative of strong negative contrast agents (Table 1).^30^ The *r*_2_ relaxivity was slightly reduced for silica coated nano-necklaces, this is likely due to the oxide coating decreasing magnetisation and reducing water access to the magnetic centres, though the signal remains strong due to the remaining strong interparticle interactions between the multiple cores comprising the entire 1-D structure. Similar strong *r*_2_ values have been observed by Sailor *et al.*, for nanoworms and Peiris *et al.*, for nanochains comprised of short chains of electrostatically-assembled superparamagnetic particles.^15,39^ A small decrease in the *r*_1_ relaxivity and subsequent significant increase in *r*_2_/*r*_1_ ratio was observed for the nano-necklaces, again attributed to the silica coating present on the surface of the nano-necklaces reducing water access to the magnetic centres as well as potentially increasing water residence times through hydrogen bonds.^40^

**Table 1:** Relaxation data collected at 23 MHz^a^

Sample	*r*_2_	*r*_1_	*r*_2_/*r*_1_
Cobalt ferrite nanoparticles	175.2 ± 3.4	7.4 ± 0.3	23.7
Silica coated nano-necklaces	129.2 ± 4.4	3.3 ± 8.6	39.2

a Error bars derived from fitting error.

Longitudinal ^1^H nuclear magnetic resonance dispersion (NMRD, Fig. 2) showed profiles typical of ferromagnetic PSSS-stabilised cobalt ferrite nanoparticles which have been reported in the literature with strong low frequency dispersion.^30,41^ The profile shape and relaxation enhancement is similar for the silica coated nano-necklaces, with this pattern reflecting nanoparticle assemblies with strong magnetic anisotropy arising from interparticle interactions (in clustered superparamagnetic species) or inherently higher magnetocrystalline anisotropy and interparticle interactions (common for ferromagnetic species), as is the case in the samples herein.^30,33,35^ The only prior example of NMRD profiles of permanently aligned 1-D nanospheres, reported by Sailor *et al.*, has shown similar behaviour, with increased low frequency dispersions for superparamagnetic nanowires.^15^ Importantly, this work, in context with other work in the literature, indicates that controlling anisotropy through structural assembly, as well as composition, can provide a means to retain strong MRI enhancement profiles, whilst also providing a readily modifiable shell and 1-D structure of use biologically.

**Fig. 2:**
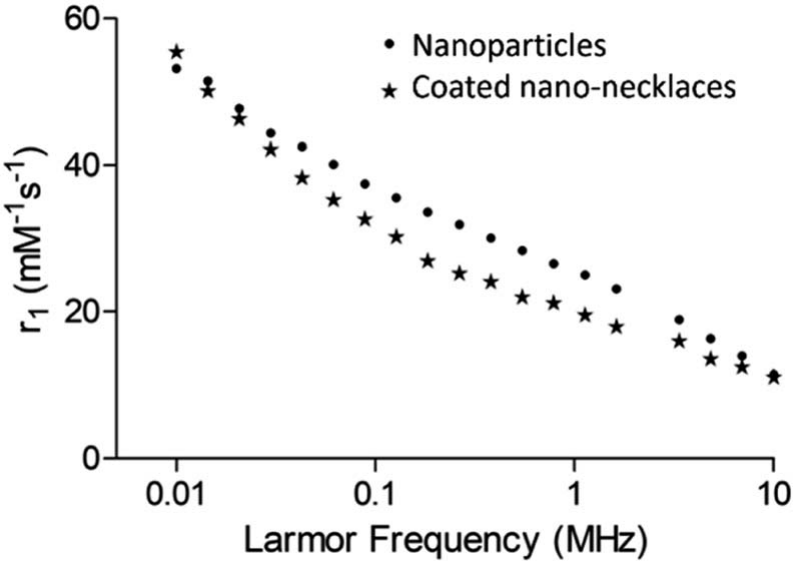
^1^H NMRD profiles measured between 0.01 and 10 MHz at 25 °C for cobalt ferrite nanoparticles and silica coated cobalt ferrite nano-necklaces.

## Conclusions

The growing importance and applications of 1-dimensional magnetic structures has led to the development of different preparative approaches in recent years. The exploitation of self-assembly between particles was amongst the first example of this, with unique and important properties identified in the resulting nanochains.^42^ Magnetically triggered assembly is the most obvious route to 1-D structures, with Sheparovych *et al.* amongst the first to showcase this route.^28^ Their elegant use of a polymer yielded ‘permanent’ 1-D arrays of ordered superparamagnetic particles. Bannwarth *et al.* took this concept further and used heat to permanently fuse aggregates of SPIONs together into chain-like structures.^43^ In our work, we sought to exploit the magnetism of 0-D particles not only to direct the formation of the 1-D structure itself, but also to drive a chemical reaction to take place at the surfaces. In this way, our approach exploits the nanoparticles themselves and provides them with a key role in forming the resulting permanent 1-D nanostructure. Our method yields 1-D nano-necklace materials which are permanently fused through a silica shell. The resulting materials exhibit good relaxation enhancement properties, with NMRD profiles typical of structures with strong structural and magnetocrystalline anisotropic behaviour resulting from permanent interparticle dipolar interactions. This behaviour is reproducible, with multiple samples exhibiting the same structures and NMRD profiles. This work therefore showcases the potential of our new approach, and illustrates the excellent properties of the resulting 1-D nanostructures for biological imaging.

## Author contributions

Aaron King: investigation, methodology, formal analysis, supervision, visualisation, writing – original draft; Teresa Insinna: investigation; Connor Wells: investigation; Isabel Raby: investigation, writing – reviewing and editing; Yurii Gun'ko: conceptualisation, writing – reviewing and editing; Gemma-Louise Davies: conceptualisation, funding acquisition, supervision, methodology, writing – reviewing and editing.

## Conflicts of interest

There are no conflicts to declare.

## Supplementary Material

NA-005-D3NA00137G-s001
