# Supplementary material for: Magnetically driven preparation of 1-D nano-necklaces capable of MRI relaxation enhancement
Source: Nanoscale Adv. 2023 May 24;5(12):3209–13. doi: 10.1039/d3na00137g (PMC10262958; doi:10.1039/d3na00137g)
Supplement: NA-005-D3NA00137G-s001 [file NA-005-D3NA00137G-s001.pdf]

Electronic Supplementary Material (ESI) for Nanoscale Advances.
This journal is © The Royal Society of Chemistry 2023

Magnetically driven preparation of 1-D nano-necklaces capable of MRI relaxation enhancement

Aaron M. King,[a] Teresa Insinna,[a] Connor Wells,[a] Isabel Raby,[a] Yurii K. Gun'ko[b] and Gemma-Louise Davies*[a]

[a] Department of Chemistry, University College London, 20 Gordon Street, London WC1H 0AJ, UK.

[b] School of Chemistry, Trinity College Dublin, Dublin 2, Ireland.

E-mail: gemma-louise.davies@ucl.ac.uk

Supporting Information

Experimental

All chemicals were used as supplied. Iron(II) chloride tetrahydrate (≥ 99 %), cobalt (II) nitrate hexahydrate (≥98 %), tetraethyl orthosilicate (TEOS, ≥99 %), poly(sodium 4-styrenesulfonate) (average Mw ~70 000), and Xanthan gum (from Xanthomonas campestris) were purchased from Sigma-Aldrich Ltd. Ammonia solution (35 % w/w) and dichloromethane (HPLC grade, ≥ 99.8 %) were purchased from Fisher Scientific, UK. Ultrapure water was collected from a Merck Milli-Q Direct water purification system operated at 18.2 MΩ. Degassed solvents were prepared through bubbling with nitrogen gas.

Preparation of PSSS-stabilised cobalt ferrite nanoparticles

$Co(NO_3)_2·6H_2O$ (0.145 g, 0.5 mmol) and $FeCl_2·4H_2O$ (0.198 g, 1 mmol) were dissolved in degassed ultrapure H_2O. Poly(sodium-4-styrenesulfonate) (PSSS, 0.05 g, 71 µmol) was dissolved in ultrapure water (10 mL) and degassed before being added to the metal salt solution and stirred to mix. NH_4OH (35 % w/w) was added in 200 µL aliquots until the pH was measured to be > 11.0. The reaction was stirred at 90 °C for 2 hours, with the black precipitate then washed with ultrapure water using centrifugation until the pH was measured neutral. The collected precipitate was then dried in air.

Preparation of silica coated cobalt ferrite nano-necklaces

Dichloromethane (DCM) (3 ml) and tetraethyl orthosilicate (TEOS) (0.9 ml, 3.6 mmol) were added to a 50 ml beaker placed on top of a permanent neodymium magnet (16.3 kg pull strength or 45.0 kg pull strength). Separately, 10 ml of aqueous PSSS-stabilised cobalt ferrite nanoparticles or non-stabilised cobalt ferrite nanoparticles (0.05 mg/ml) were combined with methanol (5 ml) and the base catalyst, NH_4OH (8.8 M). The final concentration of base in the aqueous-containing layer was 0.44 M. The aqueous-containing layer was carefully transferred *via* pipette to the beaker so that two layers would form. After 24 hours the two layers were removed and the brown precipitate at the bottom of the beaker was washed by centrifugation 3 times with ethanol and then followed by washing by magnetic separation a further 3 times. During variation reactions, final base concentration was either 2.5 M (for 'high' concentrations) or 0.09 M (for 'low' concentrations).

Characterisation

Transmission electron microscopy (TEM) images were obtained on a JEOL JEM-2100 microscope, 120 kV, operated with a beam current of 80 mA. Images were captured using a Gatan Orius 11 megapixel camera. Samples were prepared by deposition and drying of nanoparticle samples (20 μL of colloidal magnetic fluid suspensions) onto formvar-coated 300-mesh copper TEM grids (EM Resolutions). Diameters were measured using ImageJ (software version 1.8). Average values were calculated by counting a minimum of 100 particles. Magnetically aligned samples were dried in the presence of a parallel permanent magnetic field (2250 Gauss).

IR spectra were recorded using either a Bruker Alpha FTIR spectrometer or a Shimadzu IRTracer-100 FTIR spectrometer. Both machines were used in attenuated total reflectance (ATR) mode. The spectra were measured in the range 4000–400 cm^{-1}, the total number of scans and the resolution was adjusted depending on both the sample and the machine used. All samples were measured as dried solid powders unless stated otherwise. Raman spectroscopy was collected on dried solid powder samples, using a Renishaw Raman inVia microscope with a 785 nm He-Ne laser (operated at 10% equivalent to 0.76 mW). Powder X-ray diffraction was performed using a Stoe Stadi-P diffractometer with a molybdenum (Mo) X-ray source (50 kV and 30 mA), λ = 0.7093 Å. Two-theta scan range was 2–40.115 ° at a step size of 0.495 ° and 5 seconds per step. Sample holder was a transmission sample holder and samples were prepared using STOE zero scattering foils. Magnetisation measurements were carried out in the range −20.0 kOe to 20.0 kOe using a Quantum Design Physical Property Measurement System Vibrating Sample Magnetometer (VSM). The data is adjusted for the mass of sample measured to give the magnetisation in emu/g, this is based on the total mass of the solid sample, including the contribution in mass from any possible non-magnetic components.

Measurement of longitudinal ^1H nuclear magnetic resonance dispersion (NMRD) profiles were collected on a Stelar Spinmaster FFC2000 1T instrument in the range of 0.01–20 MHz Larmor frequency at two different temperatures (25 °C and 37 °C). The temperature was controlled using a Stelar VTC-91 airflow heater, equipped with a copper-constantan thermocouple; the temperature calibration in the probe head was carried out using a Delta OHM digital thermometer, with an absolute accuracy of 0.5 °C. Fast field cycling (FFC) relaxometry was used to determine the longitudinal relaxation decay over a range of relaxation fields (0.01–40 MHz). A set of 24 relaxation interval values (tau) allowed description of the spin-lattice decay curves for each relaxation field. A standard fitting algorithm (mono-exponential relaxation decay curve) allowed the evaluation of the relative longitudinal relaxation rate ($R_1 = 1/T_1$), which was converted to relaxivity using Equation (1).

Measurement of r_1 and r_2 values at a fixed field strength were carried out using an Oxford Instruments MQC+ benchtop NMR analyser with a resonant frequency of 23 MHz operated 25 °C and 37 °C. For the measurement of T_1, the standard inversion-recovery method was employed with a typical 90° pulse calibration of 250 µs with 4 scans per experiment; for T_2, the Carr-Purcell-Meiboom-Gill (CPMG) method was used with 4 scans per experiment. A minimum of 3 different concentrations of stable nanoparticle samples were prepared and relaxation time measured for each sample. r_1 and r_2 relaxivity values were calculated from curves plotted of R_1 ($1/T_1$, s^{-1}) or R_2 ($1/T_2$, s^{-1}) vs. [Fe] concentration (mM, as measured by ICP-OES) and analysis of the slope of the line of best fit for each sample, with error measured from measuring a minimum of 3 separately prepared batches of samples. Samples were dispersed in 0.5 % Xanthan gum to prevent aggregation during measurement.

An ISA Jobin Yvon Ultima 2C Inductively Coupled Plasma-Optical Emission simultaneous/sequential spectrometer (ICP-OES) running at 1 KW power with a 40.68 MHz radiofrequency Argon plasma. Plasma gas flow was 14 L min^{-1}. Nebuliser pressure was 2.6 bar at 1 mL min^{-1} sample flow rate. The spectral line for iron was measured at 259.940 nm. Samples were digested for ICP-OES using hot nitric acid and diluted in ultrapure water prior to analysis. Concentrations as measured by this technique were used to normalize all relaxation data according to Equation (1).

$$r_{1,2} = \frac{R_{1,2,obs} - R_{1,2,sol}}{[\text{total metal}]} \#(1)$$

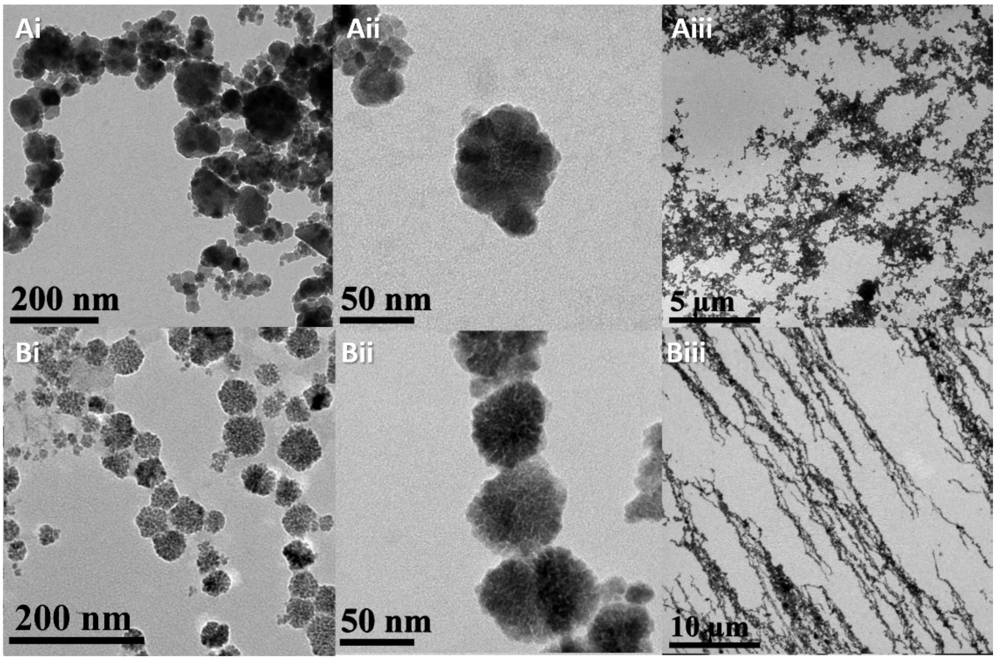

Figure S1. Transmission electron microscope (TEM) images of (A) non-stabilised $CoFe_2O_4$ particles, and (B) PSSS-stabilised $CoFe_2O_4$ particles taken (i) at low magnification, (ii) a high magnification and (iii) in the presence of an external magnetic field.

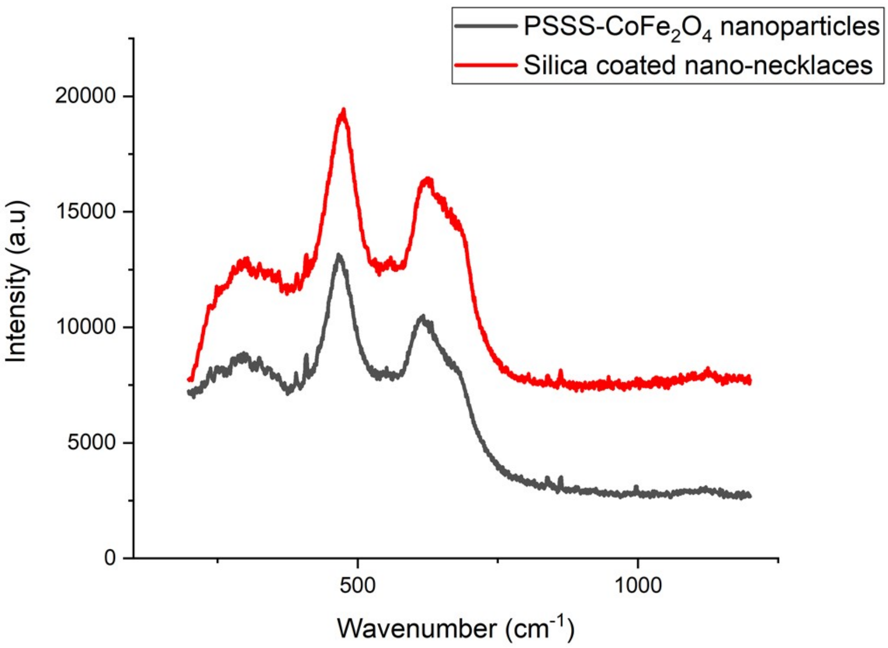

Figure S2. Raman spectroscopy with peaks at 680 cm^{-1}, 617 cm^{-1}, 475 cm^{-1}, and 300 cm^{-1} which are characteristic of the cubic inverse spinel structure of the $CoFe_2O_4$.

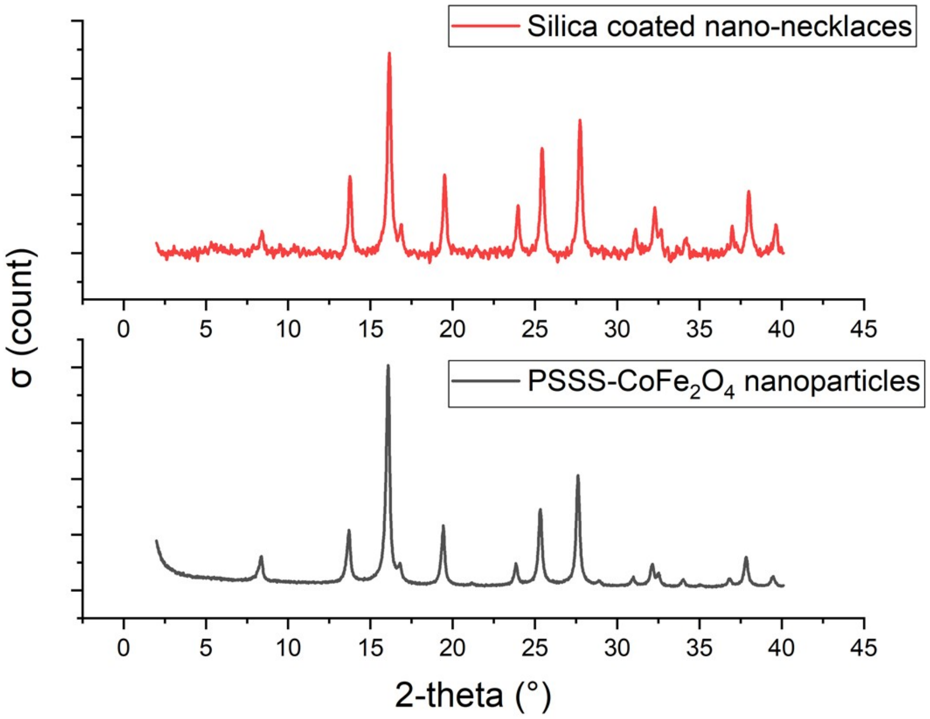

Figure S3. Powder-XRD shows reflection peaks at 13.7°, 16.1°, 19.4°, 23.8°, 25.3°, and 27.6° 2θ, indexed to (220), (311), (400), (422), (511) and (440) planes respectively, correlating well with that reported for cubic cobalt ferrite.

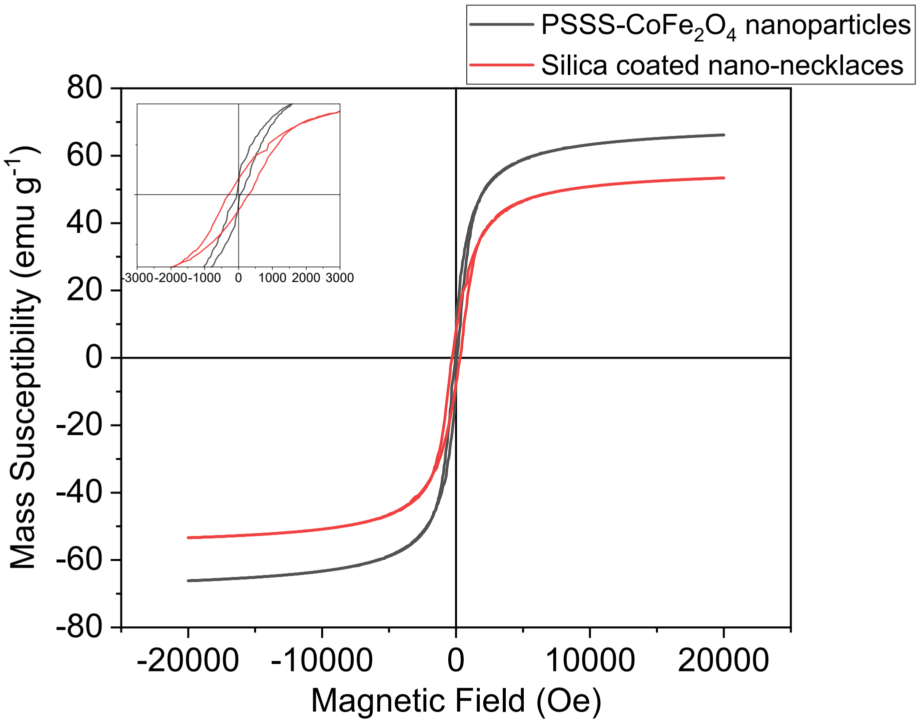

Figure S4. Vibrating sample magnetometry (VSM) of PSSS-stabilised $CoFe_2O_4$ nanoparticles (black) and corresponding silica coated nano-necklaces (red) with magnetisation saturation at 20,000 Oe of 66.2 emu/g and 53.6 emu/g, respectively.

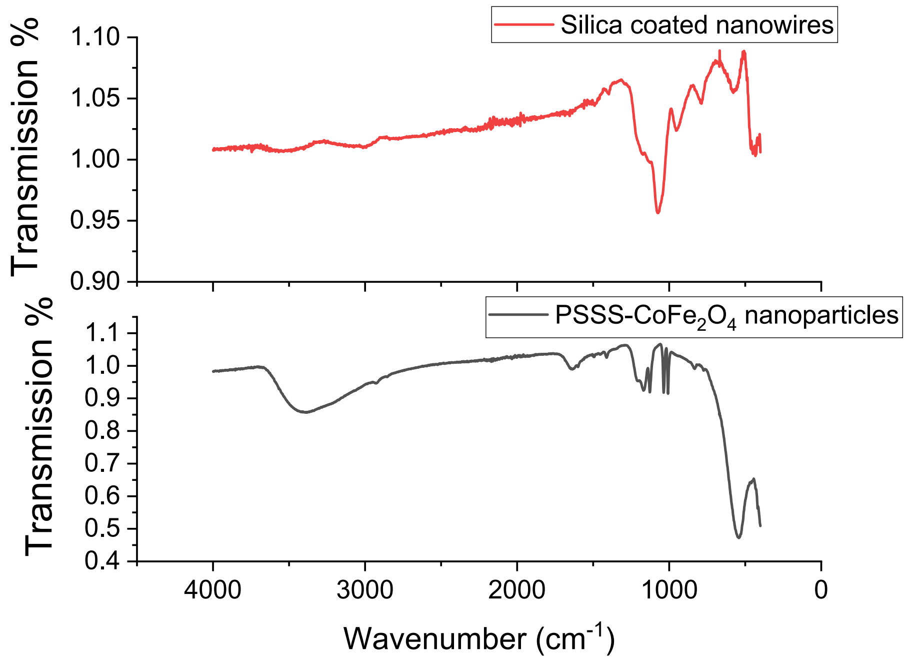

Figure S5. FTIR spectra of PSSS-stabilised $CoFe_2O_4$ nanoparticles (black) and corresponding silica coated nano-necklaces (red).

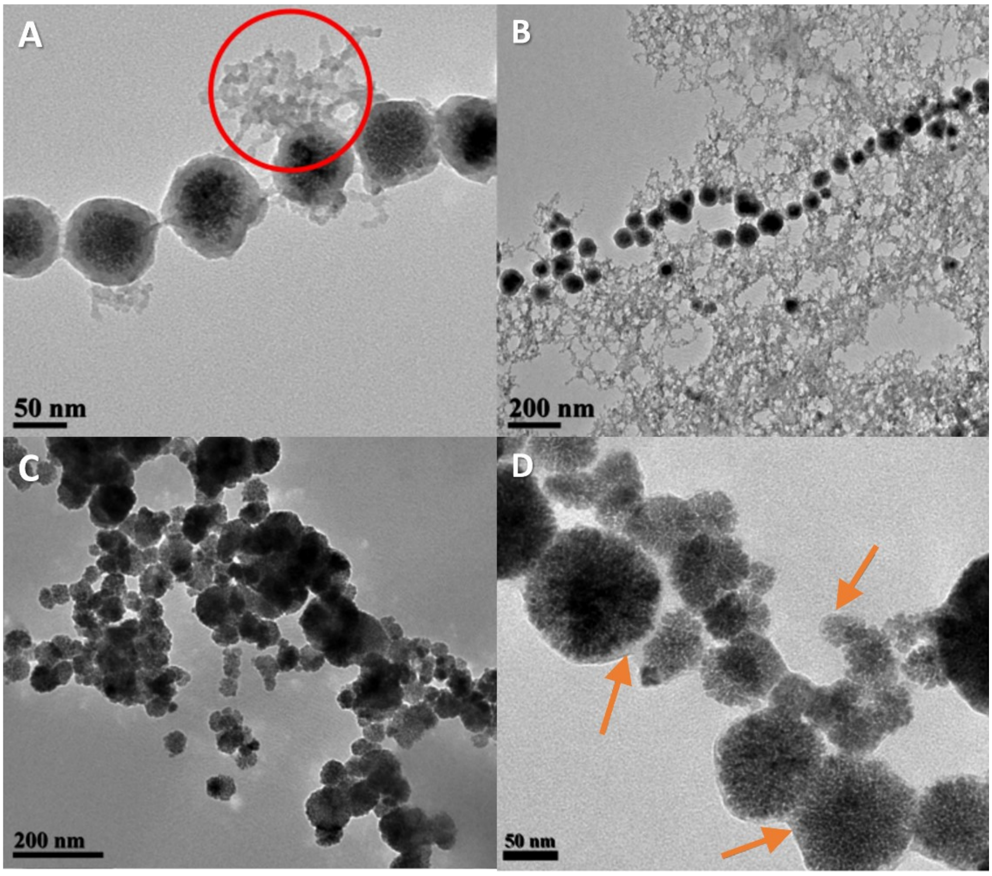

Figure S6. Transmission electron microscope (TEM) images of (A and B) nano-necklaces formed using a 'high' concentration of ammonia (2.5 M) and (C and D) nano-necklaces formed using a 'low' concentration of ammonia (0.09 M). Red circles highlight extra free silica and orange arrows show thin silica shells around nanoparticles.

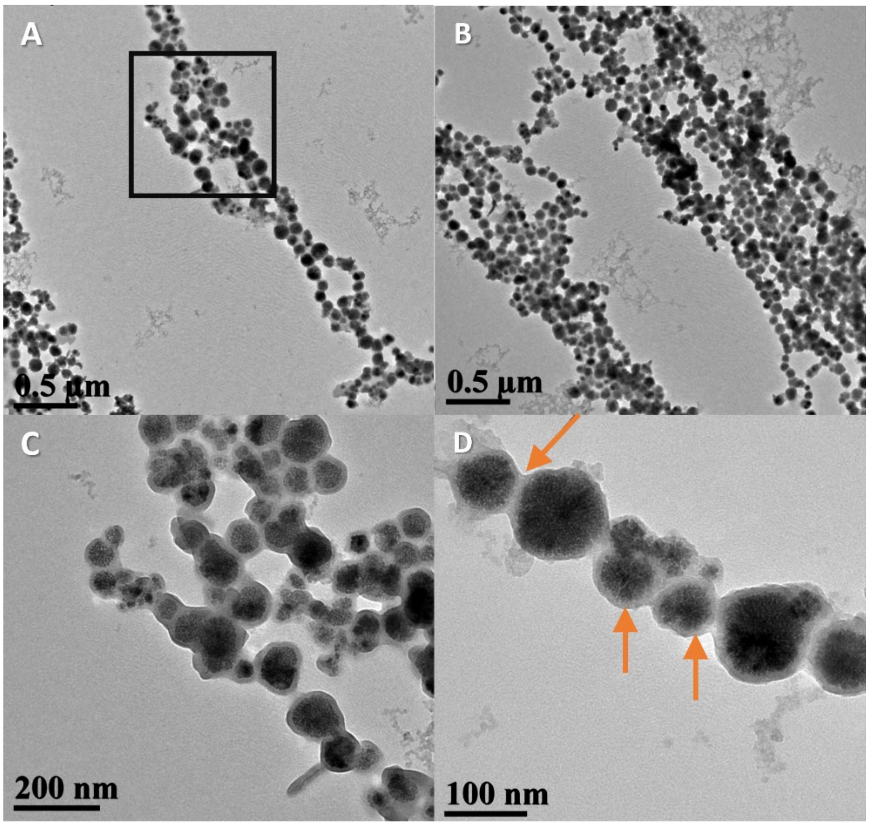

Figure S7. Transmission electron microscope (TEM) images of nano-necklaces with thicker 'bundles' of chains formed using a stronger (45.0 kg pull strength) magnet during preparation. Image (c) shows higher magnification of the box highlighted in (a). Orange arrows show silica coating.
